# Supplementary material for: Miniaturized spectrometer with intrinsic long-term image memory
Source: Nat Commun. 2024 Jan 23;15:676. doi: 10.1038/s41467-024-44884-1 (PMC10805890; doi:10.1038/s41467-024-44884-1)
Supplement: Supplementary file 1 — Supplementary Information [file 41467_2024_44884_MOESM1_ESM.pdf]

Supplementary Information

Miniaturized spectrometer with intrinsic long-term image memory

Gang Wu[1], Mohamed Abid[1], Mohamed Zerara[2], Jiung Cho[3,4], Miri Choi[5], Cormac Ó Coileáin[6], Kuan-Ming Hung[7], Ching-Ray Chang[8,9], Igor V. Shvets[10], and Han-Chun Wu[1,]*

[1]School of Physics, Beijing Institute of Technology, Beijing 100081, P. R. China

[2]University of Applied Sciences, Geneva, Switzerland

[3]Western Seoul Center, Korea Basic Science Institute, Seoul 03579, Republic of Korea

[4]Department of Advanced Materials Engineering, Chung-Ang University, 4726, Seodong-daero, Daedeok-myeon, Anseong-si, Gyeonggi-do, 17546, Republic of Korea

[5]Chuncheon Center, Korea Basic Science Institute, Chuncheon 24341, Republic of Korea

[6]Institute of Physics, Faculty of Electrical Engineering and Information Technology, University of the Bundeswehr Munich, Neubiberg 85577, Germany

[7]Department of Electronics Engineering, National Kaohsiung University of Science and Technology, Kaohsiung 807, Taiwan, ROC

[8]Quantum information center, Chung Yuan Christian University, Taoyuan 32023, Taiwan, ROC

[9]Department of Physics, National Taiwan University, Taipei 106, Taiwan, ROC

[10]School of Physics, Trinity College Dublin, Dublin, Dublin 2, Ireland

*Address correspondence to: wuhc@bit.edu.cn

Supplementary Table 1. Comparison between this work and other miniaturized spectrometers.

Materials/Structures	Spectral range (nm)	Footprint (μm)	Resolution (nm)	Ref.
$SnS_2/ReSe_2$ vdW heterostructure	400 ~ 800	19	5	This work
MoS_2/WSe_2 vdW heterojunction	405 ~ 845	22	3	1
$ReS_2/Au/WSe_2$ vdW heterostructure	1150 ~ 1470	6	20	2
Black phosphorus	2000 ~ 9000	16	90	3
Single nanowire	500 ~ 630	75	10	4
Quantum dots	400 ~ 700	8500	3.2	5
Photonic Crystal	550 ~ 750	210	2	6
Structurally colored nanowires	450 ~ 800	2000	6	7
Interferometer	1500 ~ 1600	512	4	8
Folded metasurfaces	760 ~ 860	7000	1.2	9
Perovskite	450 ~ 780	5600	80	10
Spiro-OMeTAD/Perovskite	350 ~ 750	440	30	11
Voronoi photonic crystal	420 ~ 720	233	10	12
Quantum dots	400 ~ 2000	100	13	13
Thin-film lithium niobate	1040 ~ 1550	5000	5.5	14
Disordered photonic chip	1500 ~ 1525	100	0.75	15
Holographic	719 ~ 861	1000	0.145	16
Ring resonator	1460 ~ 1610	200	1	17

Supplementary Note 1: Photogating effect induced by defects

In order to comprehend the photogating effect induced by defects in the heterostructure, we performed TEM analysis and XPS characterization, and also conducted first-principle calculations. **Figs. 1c** and **1d** of the main text show high-resolution TEM images of mechanically exfoliated SnS_2 and $ReSe_2$ layers. One can see that both exfoliated nanosheets are highly crystalline in nature. However, some vacancies could be observed (red arrows). To determine the type and estimate the concentration of vacancies, X-ray photoelectron spectroscopy (XPS) was carried out on SnS_2 and $ReSe_2$ to ascertain both their different oxidation states and stoichiometry, crucial factors in determining their fundamental properties, which can influence the device working principle. **Supplementary Figs**. **6a** and **6b** show the core level spectra for Sn 3*d* and S 2*p*, respectively. The binding energy values were calibrated with reference to the carbon 1*s* peak, which was set at 284.7 eV. The Sn $3d_{5/2}$ transition was deconvoluted into two values at binding energy (BE) values of 485.3 eV and 486.4 eV, corresponding to the Sn oxidation state of +2 and +4. The sulfur 2*p* core-level XPS spectrum showed two peaks at BEs of 162.7eV and 163.7 eV. Finally, the atomic weight percentages obtained from the deconvoluted states reveal a stoichiometry of $SnS_{1.98}$, indicating ~2 % sulfur vacancies in our SnS_2. **Supplementary Figs**. **6c** and **6d** depict the core-level spectrum of Re 4*f* and Se 3*d*. In the case of the Re 4*f* core-level spectrum, it typically exhibits two peaks corresponding to Re $4f_{7/2}$ and Re $4f_{5/2}$, with binding energy values of 42.1 eV and 44.5 eV, respectively. For the core-level XPS spectra of Se 3*d*, to obtain a satisfactory fitting, four peaks were used, instead of two, corresponding to Se $3d_{5/2}$ and Se $3d_{3/2}$. Furthermore, the atomic weight percentages obtained from the deconvoluted states reveal a stoichiometry of $ReSe_{2.04}$, indicating ~2.4 % Re vacancies. The spectrum complexity and the additional peak observed may be related to the production of electron-deficient $Se^{(2-\delta)-}$ sites in our Se-rich $ReSe_{2+x}$ materials.

Supplementary Figs. 7a-7d show the atomic structures for the first-principle calculations. We considered a 2x2x2 super cell structure for ideal $ReSe_2$ and a 3x3x2 super structure for ideal SnS_2. To investigate the effect of Re vacancies and S vacancies,

one Re atom and one S atom were removed from the ideal $ReSe_2$ and $ReSe_2$ structures, respectively. **Supplementary Figs. 7e-7h** plots the corresponding calculated electronic structures. Note, the top of the valance bands (VB) was shifted to zero. Interestingly, when one Re atom was removed, three defect bands appeared and were labeled as DF_{R1} (0.26 eV below bottom of conduction band (CB)), DF_{R2} (0.49 eV below the bottom of CB), and DF_{R3} (0.37 eV above the top of VB). For SnS_2 with a S vacancy, only one defect band appeared (DF_S) and is located 0.7 eV below the bottom of the CB.

To further understand the defect induced photogating effect in the heterostructure, we calculated the Fermi energies for SnS_2, $ReSe_2$, and a $SnS_2/ReSe_2$ heterostructure. **Supplementary Figs. 8a** and **8b** show the transfer characteristics of SnS_2 and $ReSe_2$ respectively. The extrinsic field-effect mobility, μ_{FE}, of the electrons in SnS_2 and $ReSe_2$ can be calculated from the equation $\mu_{\mathrm{FE}} = \frac{g_{\mathrm{m}} L}{W C_{\mathrm{ox}} V_{\mathrm{ds}}}$ at a constant drain-source voltage V_{ds} = 2 V, where $g_{\mathrm{m}} = \frac{\partial I_{\mathrm{ds}}}{\partial V_{\mathrm{g}}}|_{V_{\mathrm{ds}}=\mathrm{const}}$ is the transconductance of the field effect transistor, L is channel length (20 μm for both SnS_2 and $ReSe_2$), W is the channel width (27 μm for SnS_2 and 36 μm for $ReSe_2$), C_{ox} = 5.76×10^{-5} F/m^2 is the capacitance of the gate oxide (300 nm thick SiO_2). Using the slope, *i.e.* g_{m}, obtained from the linear fit to the linear region of the transfer curve, we can get the $\mu_{\mathrm{FE}}{}^{\mathrm{SnS_2}}$ = 2.17 cm$^2\cdot$(V·s)$^{-1}$, $\mu_{\mathrm{FE}}{}^{\mathrm{ReSe_2}}$ = 0.614 cm$^2\cdot$(V·s)$^{-1}$. Using the calculated mobilities, we can obtain the Fermi energies for SnS_2 (-4.38 eV) and $ReSe_2$ (-4.03 eV).

The Fermi energy of the $SnS_2/ReSe_2$ heterostructure was determined by requiring that the increased charges in the SnS_2 equaled the decrease in $ReSe_2$, for the initial Fermi level of $ReSe_2$ being higher than that in SnS_2, i.e.,

$$\Delta n_{\mathrm{e}} = n_{\mathrm{e0S}}(E_{\mathrm{F0S}}) - n_{\mathrm{eS}}(E_{\mathrm{F}}) = -[n_{\mathrm{e0R}}(E_{\mathrm{F0R}}) - n_{\mathrm{eR}}(E_{\mathrm{F}})], \quad (1)$$

where $n_{\mathrm{e0S(R)}}$ is the initial electron density of SnS_2 ($ReSe_2$) with the initial Fermi energy $E_{\mathrm{F0S(R)}}$, and $n_{\mathrm{eS(R)}}$ is the electron density after charge transfer from $ReSe_2$ into SnS_2 with the Fermi energy E_{F}. The Fermi energy E_{F} and the transfer charge can be estimated by the following expressions

$$E_F = E_{F0R} + k_B T \times \ln\left(1 + \frac{\Delta n_e}{n_{e0R}}\right) \text{ and} \quad (2)$$

$$\Delta n_e = n_{e0S}(E_{F0S}) - N_{CS} e^{-\frac{E_{CS}-E_F}{k_B T}}, \quad (3)$$

N_{CS} is the effective conduction-band density of states, it takes the value $4.615 \times 10^{26}\ m^{-3}$, and E_{CS} $(-4.22eV)$ is the conduction band edge of SnS_2. Solving Equations 1-3 yields a Fermi energy of -4.35 eV. The built-in potentials V_{biS} and V_{biR} at SnS_2 and $ReSe_2$ are calculated by $V_{biS} = E_F - E_{F0S}$ and $V_{biR} = E_{F0R} - E_F$. The calculated results are shown in **Supplementary Fig. 8e**. The largest built-in potential is distributed on the $ReSe_2$ side, however, the SnS_2 side changes the most with increasing V_g. Moreover, the energy band of $ReSe_2$ bends upward and that of SnS_2 bends downward after contact. **Supplementary Fig. 8f** shows a schematic of the energy bands of the SnS_2/$ReSe_2$ heterostructure after contact. One can find that for SnS_2, DF_S is located below the Fermi energy and far above the VB of SnS_2. Thus, traps in the SnS_2 can be ignored. However, for $ReSe_2$, the energy distance between the bottom of DF_{R3} and the top of the VB is only ~ 0.07 eV. Moreover, due to the upward bending of the energy band, hole trapping plays a unique role in the overlapping region as excited holes will move to the overlapped region and electrons will move to the nonoverlapped region. Thus, under illumination, holes are excited and move to the overlapping region and some of the holes will be trapped by DF_{R3}, resulting a photogating effect, which would have the effect of enhancing the photocurrent. When the light is switched off, the trapped holes remain and sustain the photocurrent, resulting in a memory effect. We would like to stress that the number of holes trapped depends on V_{biR}. Increasing V_g, V_{biR} decreases, resulting in a decrease in the number of trapped holes, which is consistent with the experimental results. For photodetectors dominated by a photogating effect, the photocurrent (I_{ph}) can be written as $I_{ph} = \frac{\partial I_{ds}}{\partial V_g} \Delta V_g$, where ΔV_g is the local gate voltage generated by photoexcited carrier trapping at the interface. In **Fig. 2f** of main text, the calculated ΔV_g is shown to decrease with increasing gate voltage.

Moreover, the interfacial trap states, such as neutral traps (NT), are generally observed in 2D heterostructures during stacking process, The upwardly bent band raises the

energy position of NTs in the overlapping region above the Fermi level (**Supplementary Fig. 9a**). Under illumination, electrons of the NTs in the overlapping region are excited to the conduction band and transported to the lower energy side (non-overlapping SnS_2 region) as shown in **Supplementary Fig. 9b**. In this case, the NTs become positively charged, lowering the transport barrier at the interface and thus increasing the photocurrent. When the light is switched off, the partially charged NTs remain at a position above the Fermi level, thereby sustaining the photocurrent, again producing a memory effect. Thus, neutral traps at the interface also result in photogating and memory effects.

Supplementary Note 2: Photocurrent generated in SnS_2/$ReSe_2$ heterostructure

The total photocurrent (I_{ph}) has three main contributions: the photocurrents generated in the $ReSe_2$ (I_{R}) and SnS_2 regions (I_{S}), and the photocurrent generated in the overlapping region ($I_{interface}$) due to the photogating effect. As the Au electrodes have a much greater work function compared with those of SnS_2 and $ReSe_2$, built-in potential between Au and $ReSe_2$ (ϕ_{B1}) is greater than V_{biR}. Thus, I_{S} and I_{R} are in opposite directions, having opposing contributions. We can further write the photocurrents generated in the ReS_2 and SnS_2 regions as: $I_i = qG_i(\lambda)\tau_i\mu_i SV_{\mathrm{bi}i}/W_{\mathrm{D}i}$, where I_i, $G_i(\lambda), \tau_i$, μ_i, $V_{\mathrm{bi}i}$, and $W_{\mathrm{D}i}$ are the photocurrent, photogeneration rate, lifetime, mobility, effective built-in potential, and effective depletion width in region *i*, respectively. The built-in potential of the SnS_2 side decreases the most with increasing V_{g} and that in the $ReSe_2$ side remains quite stable (**Supplementary Fig. 8e**). In other words, the photocurrent decreases much faster with increasing V_{g}. Thus, the peak position of the photocurrent moves to longer wavelengths with increasing V_{g}, which is consistent with the experimental observations (**Fig. 2f**).

Supplementary Note 3: Deriving the Nonlinearity of the LTP and LTD

In order to achieve this, we used an asymmetric nonlinear relationship to fit the LTP (1) and LTD (2) curves, thereby deriving the nonlinearity (NL) and the weight change of LTP and LTD during the weight update process.

$$I_{\mathrm{P}}(N) = I_{\mathrm{m\,in}} + B \times \left(1 - e^{\left(-\frac{N}{A_{\mathrm{P}}}\right)}\right) \qquad (4)$$

$$I_{\mathrm{D}}(N) = I_{\max} - A \times \left(1 - e^{\left(\frac{N-N_{\max}}{A_{\mathrm{D}}}\right)}\right) \qquad (5)$$

$$B = (I_{\max} - I_{\min})\left(1 - e^{\left(-\frac{N_{\max}}{A_{\mathrm{P,D}}}\right)}\right) \qquad (6)$$

where I_{P} and I_{D} are functions describing the current of the potentiation and depression curves, respectively. $I_{\max}$, $I_{\min}$ and N represent the maximum current, minimum current, and number of applied pulses, respectively. Furthermore, the non-linearity is quantitatively determined by using the following the relation (7):

$$\mathrm{ANL} = \left[\frac{I_{\mathrm{P}}(N/2) - I_{\mathrm{D}}(N/2)}{I_{\max} - I_{\min}}\right]. \qquad (7)$$

Potentiation and depression curves that are entirely symmetric result in an ANL (Asymmetry of Nonlinearities) value of zero, whereas our device exhibits an ANL value of 0.35.

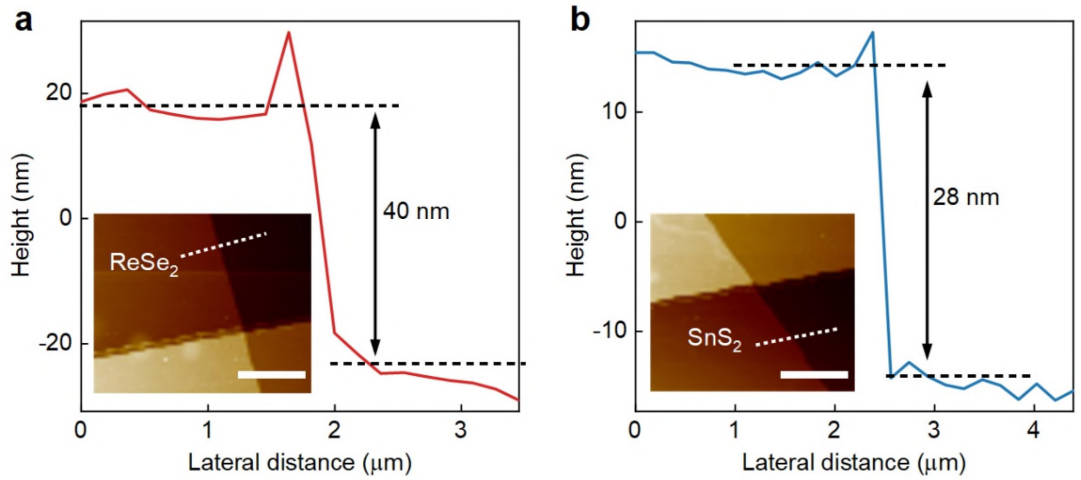

Supplementary Fig. 1 | Atomic force microscopy (AFM) characterization of $ReSe_2$ and SnS_2. Thicknesses of **(a)** $ReSe_2$ and **(b)** SnS_2 layers, measured using an atomic force microscope, determined to be 40 nm and 28 nm, respectively. The insets show the corresponding AFM height-map images. Scale bar, 2 μm.

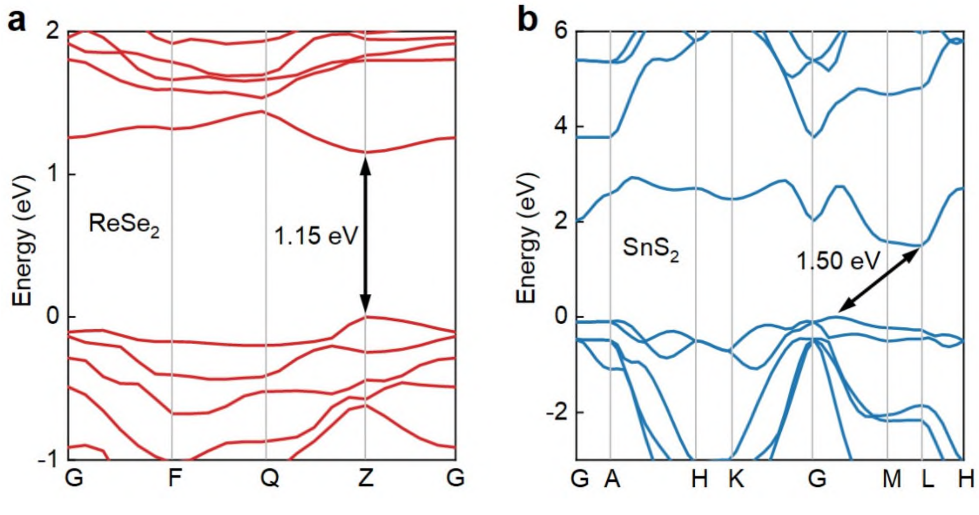

Supplementary Fig. 2 | Band structures of $ReSe_2$ and SnS_2. Calculated band structure of **(a)** $ReSe_2$ and **(b)** SnS_2 with bandgaps of 1.15 eV and 1.50 eV, respectively.

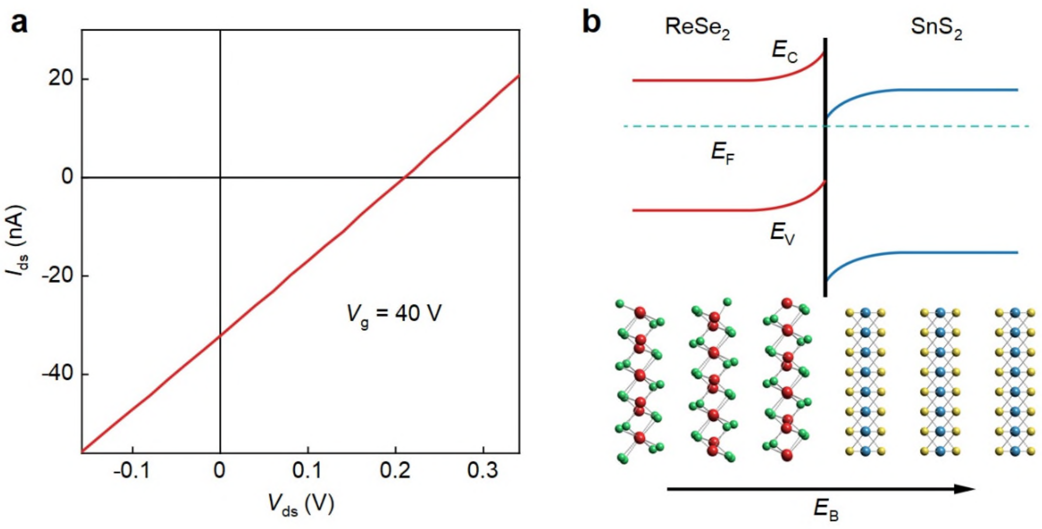

Supplementary Fig. 3 | Electrical characterization of the band alignment of the device. (a) I_{ds}-V_{ds} curve of the $ReSe_2$ / SnS_2 device under 635 nm laser illumination (P_{in} = 20 mW/cm^2) at V_g = 40 V, showing an open-circuit voltage (V_{OC}) of ~0.22 V and a short-circuit current (V_{SC}) of ~32 nA. **(b)** Band alignment of the heterostructure at the interface. The arrow indicates the direction of the built-in electric field (E_B).

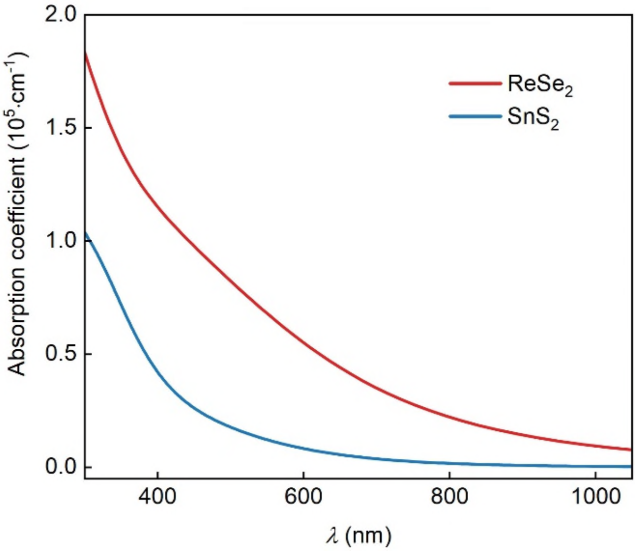

Supplementary Fig. 4 | Calculated absorption coefficients of bulk $ReSe_2$ and SnS_2.

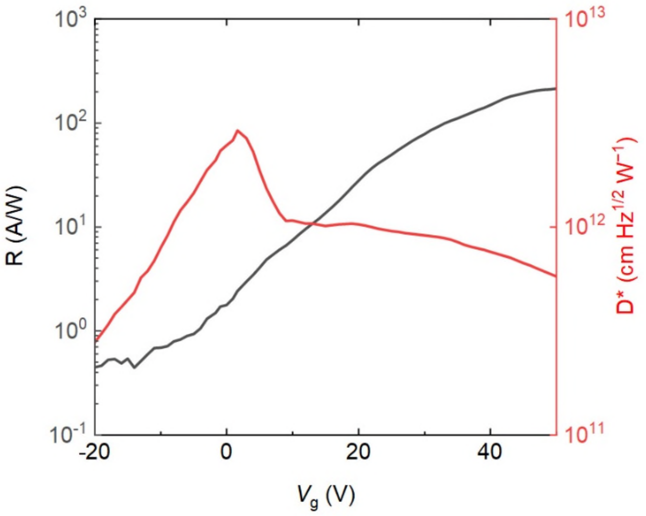

Supplementary Fig. 5 | Photodetection performance of the device. The responsivity (*R*, black line) and specific detectivity (D*, red line) of the $ReSe_2$ / SnS_2 device under 635 laser illumination (P_{in} = 0.22 mW/cm^2) as a function of gate voltage.

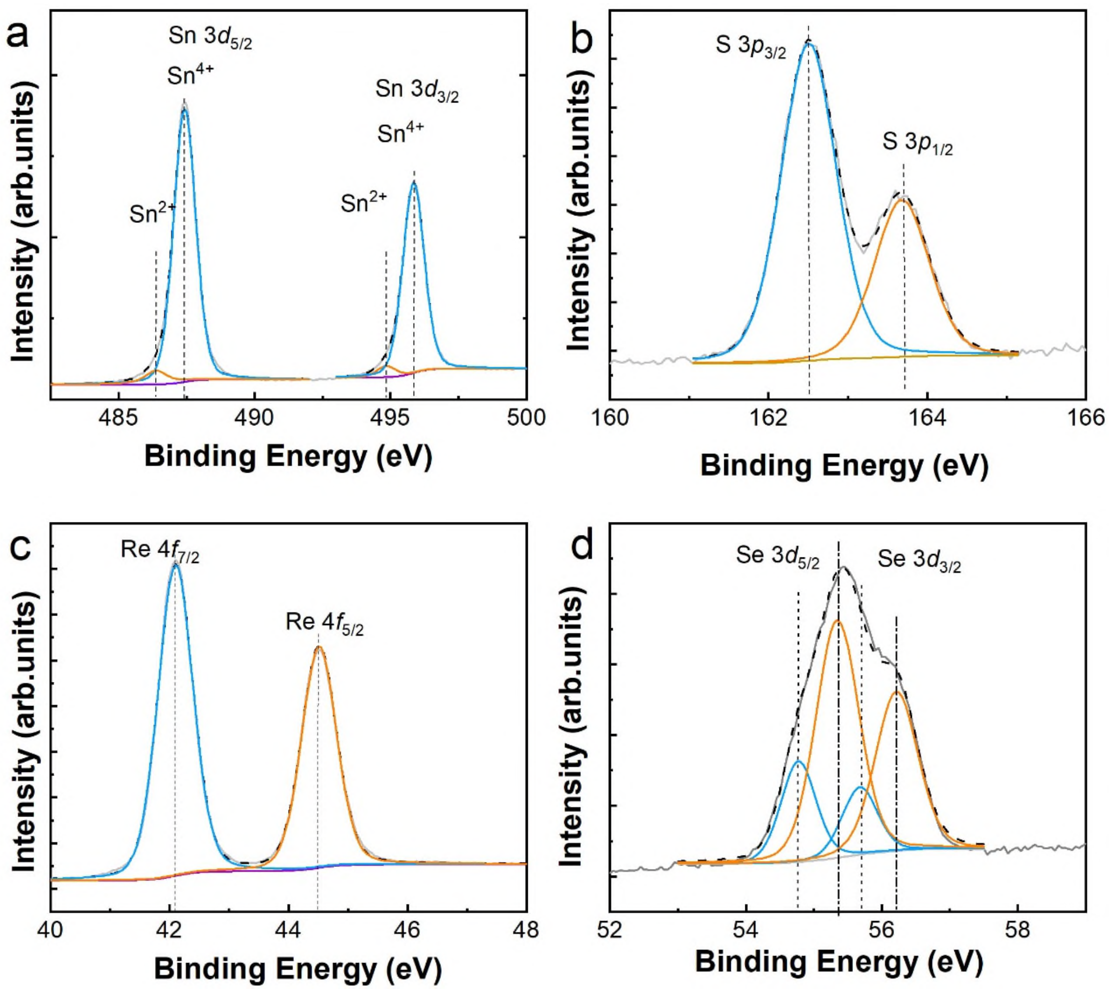

Supplementary Fig. 6 | X-ray photoelectron spectroscopy (XPS) characterization of $ReSe_2$ and SnS_2. (a) High-resolution XPS spectra of Sn 3$d_{3/2}$ and Sn 3$d_{5/2}$, (b) S 2$p_{1/2}$ and S 2$p_{3/2}$ region of SnS_2. (c) High-resolution XPS spectra of Re 3$f_{7/2}$ and Re 3$f_{5/2}$, (d) Se 3$d_{5/2}$ and Se 3$d_{3/2}$ region of $ReSe_2$.

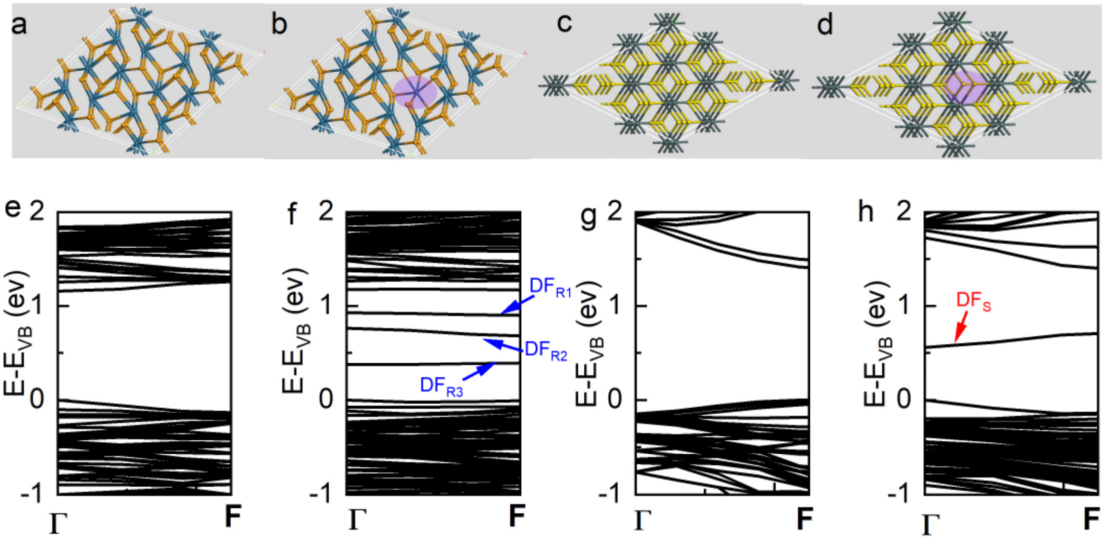

Supplementary Fig. 7 | Defect band calculations of $ReSe_2$ and SnS_2. (a) Atomic structures of ideal $ReSe_2$, (b) $ReSe_2$ with Re vacancy, (c) ideal SnS_2, and (d) SnS_2 with S vacancy, respectively. (e) Electronic structures of ideal $ReSe_2$, (f) $ReSe_2$ with Re vacancy, (g) ideal SnS_2, and (h) SnS_2 with S vacancy, respectively.

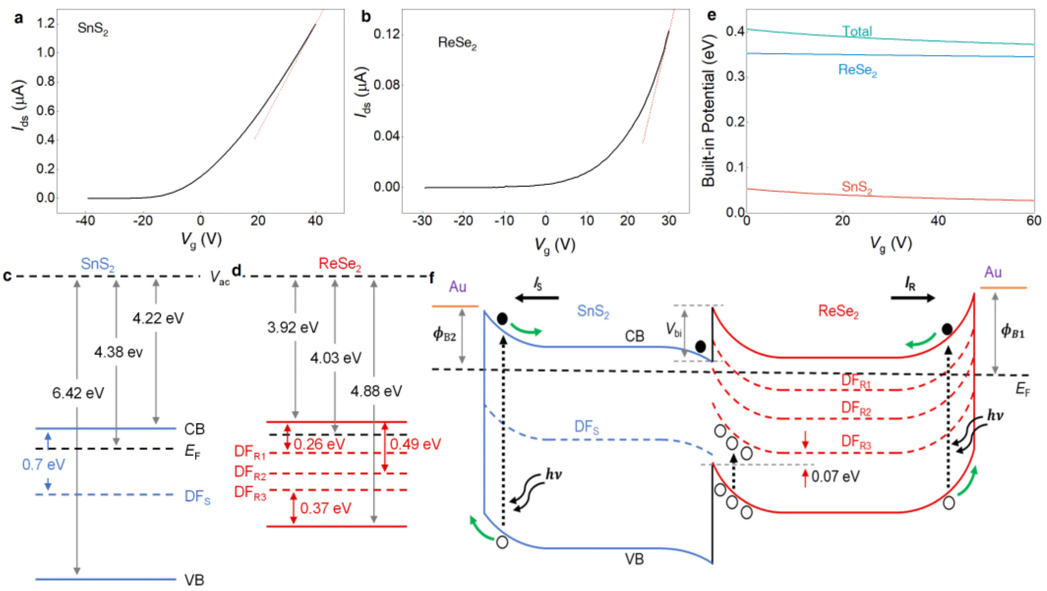

Supplementary Fig. 8 | Band profile of $ReSe_2$, SnS_2 and their combined heterostructure. Transfer characteristics of (a) SnS_2 and (b) $ReSe_2$ in darkness at V_{ds} = 2 V. (c) Schematic of the electronic structure of SnS_2 and (d) $ReSe_2$. (e) Calculated built-in potential of the total heterostructure (V_{bihet}, green line), $ReSe_2$ (V_{biR}, blue line) and SnS_2 (V_{biS}, red line). (e) Schematic of energy band of SnS_2/$ReSe_2$ heterostructure after contact.

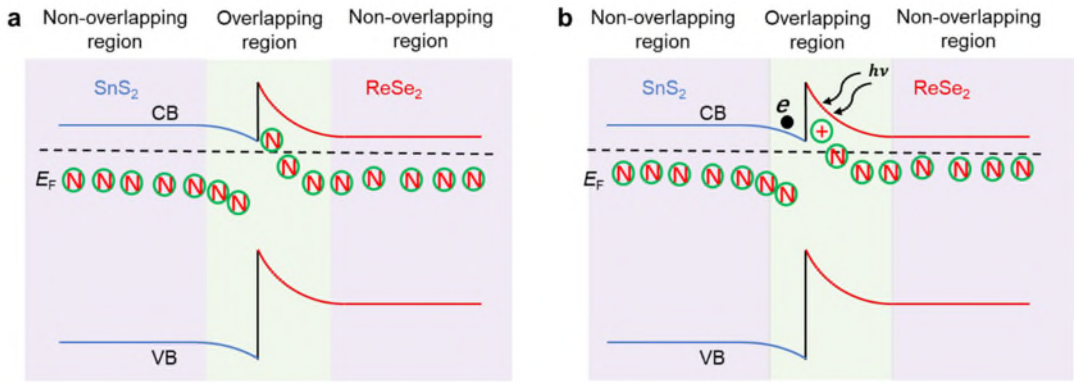

Supplementary Fig. 9 | Schematic diagram of the heterojunction band profile (a) before and (b) after illumination.

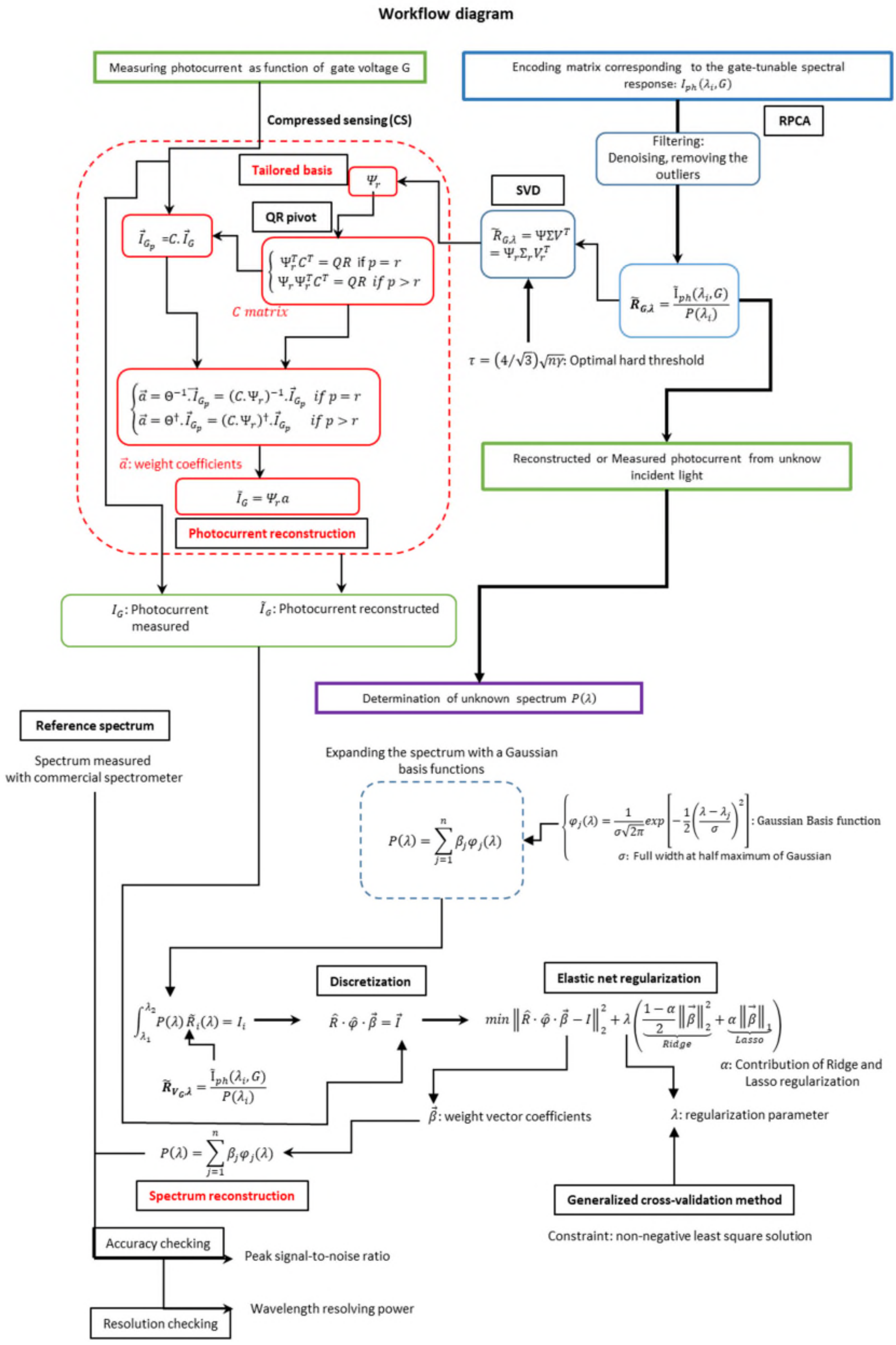

Supplementary Fig. 10 | Workflow of the spectrometer. Summarized workflow diagram showing the detailed spectrum reconstruction process for our single-$SnS_2/ReSe_2$-heterostructure spectrometer.

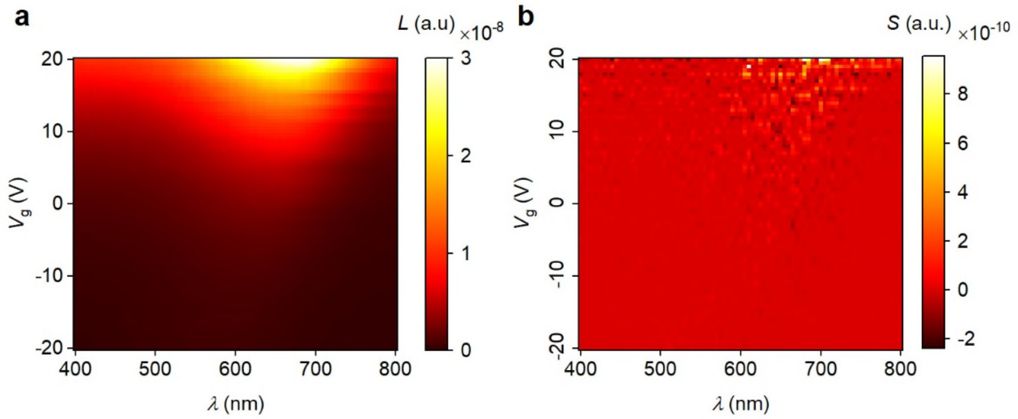

Supplementary Fig. 11 | Decomposition of spectral matrix responsivity $\boldsymbol{R_{G,\lambda}}$ into (a) a low-rank matrix $\boldsymbol{\widetilde{R}_{G,\lambda}}$ and (b) a sparse matrix $\boldsymbol{S_{G,\lambda}}$.

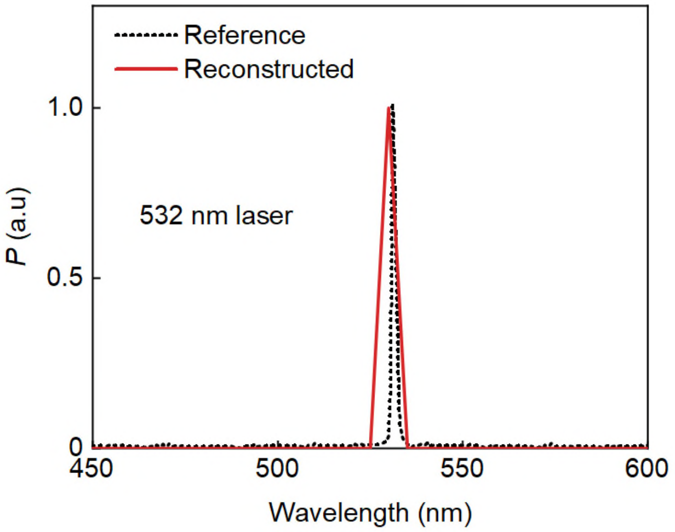

Supplementary Fig. 12 | 532 nm laser spectrum reconstruction. The reconstructed 532 nm laser spectrum using optimized compressed sensing method and the reference spectrum measured using a commercial spectrometer.

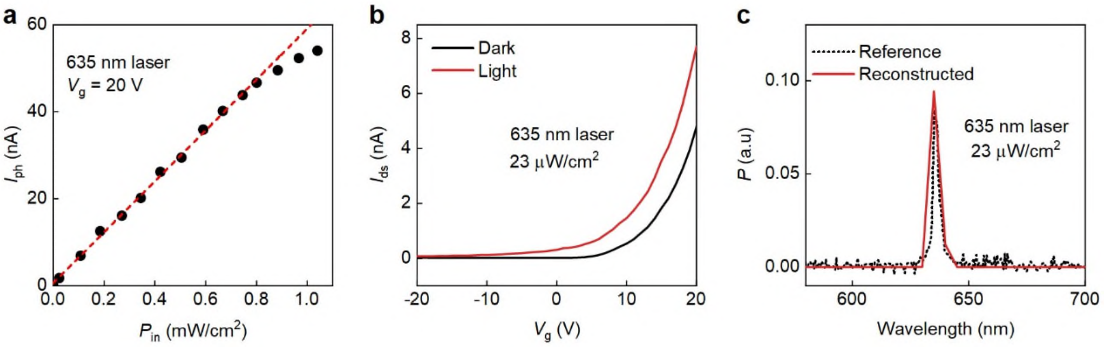

Supplementary Fig. 13. | Dynamic range of the spectrometer. (a) Photocurrent of the device as a function of 635 nm laser power density at V_g = 20 V. **(b)** Transfer curves of the device in dark conditions (black) and under 635 nm laser illumination (P_{in} = 23 μW/cm^2, red). **(c)** Reconstructed 635 nm laser spectrum and the reference spectrum measured using a commercial spectrometer.

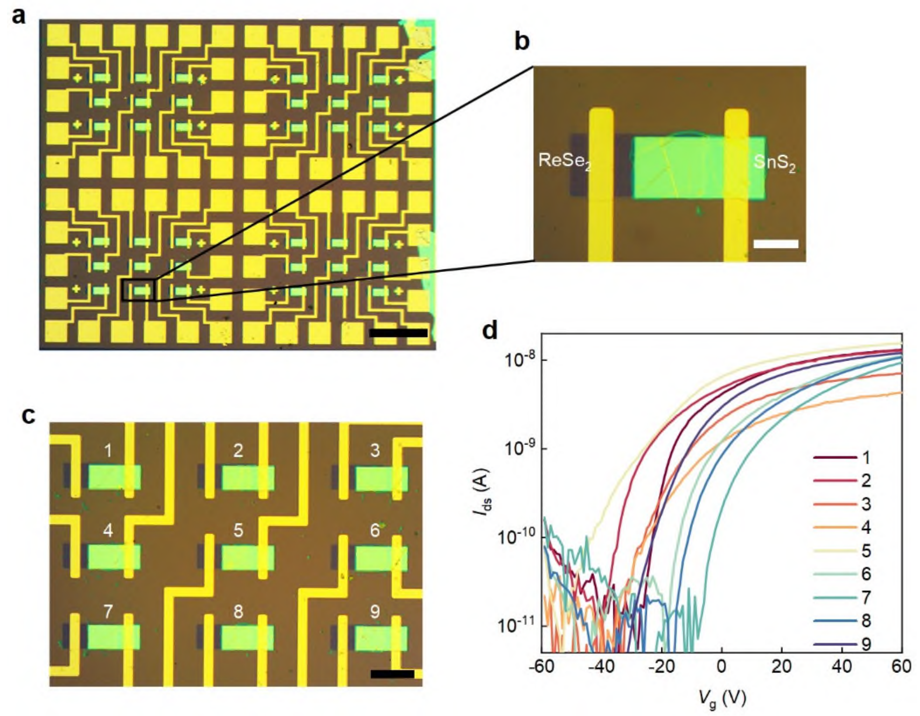

Supplementary Fig. 14 | Characterization of SnS_2/$ReSe_2$ heterostructure array. (a, b, c) Optical microscopy images of the 3×3 SnS_2/$ReSe_2$ heterostructure array. Scale bar, **(a)** 200 μm, **(b)** 20 μm, **(c)** 50 μm. **(d)** Transfer curves of devices shown in **(c)**.

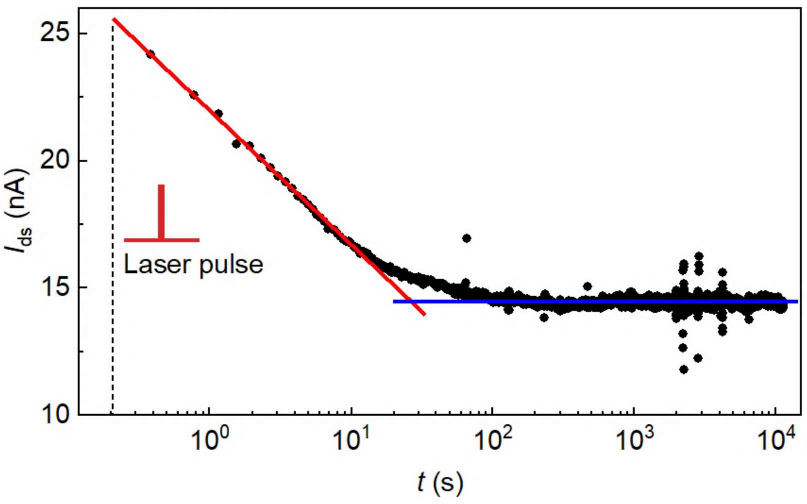

Supplementary Fig. 15 | Long term decay behavior of photocurrent. Time resolved source-drain current of the device after applying a program laser pulse (P_{in} = 12 mW/cm^2, pulse-width ~0.1 s) at V_{ds} = 1 V and V_g = 0. It is found that there is a transition from logarithmic decay to a nearly relaxation-free state, indicated by the solid lines.

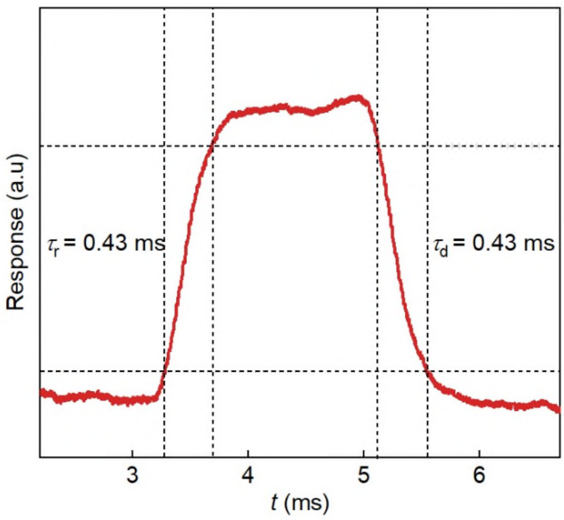

Supplementary Fig. 16 | Response time measurement. Response time measurement of the ‘fast mode’, showing a rise time and a decay time for both of 0.43 ms.

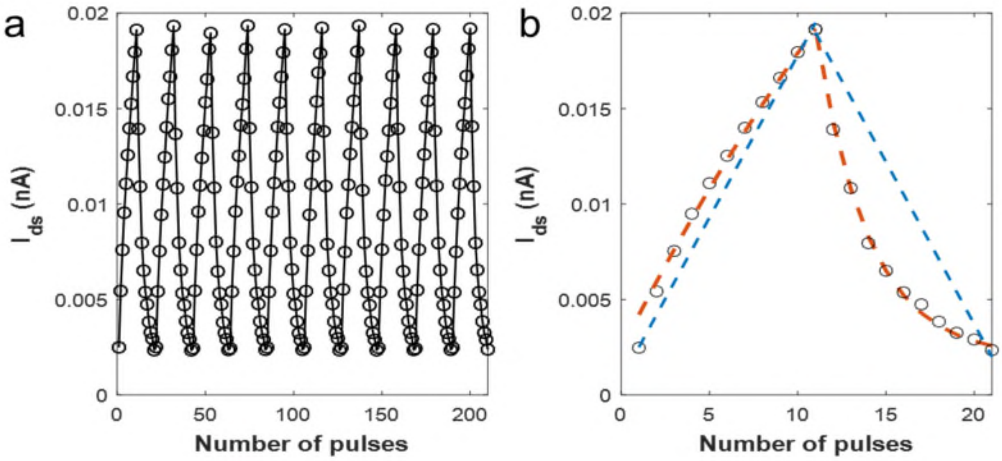

Supplementary Fig. 17 | Long-term potentiation (LTP) and depression (LTD) cycles. (a) LTP and LTD curves of the vdW $SnS_2/ReSe_2$ device for 10 cycles. The excitatory pulse is a blue light (pulse-width ~1 s) with P_{in} ~10 mW/cm^2, whereas the inhibitory V_g is a pulse with an amplitude of +5 V. (b) A single LTP/LTD curve of vdW $SnS_2/ReSe_2$ synaptic device, where the asymmetric nonlinearity factor (ANL) is of 0.35.

Supplementary References

1 Yoon, H. H. *et al.* Miniaturized spectrometers with a tunable van der Waals junction. *Science* **378**, 296–299 (2022).

2 Deng, W. *et al.* Electrically tunable two-dimensional heterojunctions for miniaturized near-infrared spectrometers. *Nat. Commun.* **13**, 4627 (2022).

3 Yuan, S., Naveh, D., Watanabe, K., Taniguchi, T. & Xia, F. A wavelength-scale black phosphorus spectrometer. *Nat. Photonics* **15**, 601–607 (2021).

4 Yang, Z. *et al.* Single-nanowire spectrometers. *Science* **365**, 1017–1020 (2019).

5 Bao, J. & Bawendi, M. G. A colloidal quantum dot spectrometer. *Nature* **523**, 67–70 (2015).

6 Wang, Z. *et al.* Single-shot on-chip spectral sensors based on photonic crystal slabs. *Nat. Commun.* **10**, 1020 (2019).

7 Meng, J., Cadusch, J. J. & Crozier, K. B. Detector-only spectrometer based on structurally colored silicon nanowires and a reconstruction algorithm. *Nano Lett.* **20**, 320–328 (2020).

8 le Coarer, E. *et al.* Wavelength-scale stationary-wave integrated Fourier-transform spectrometry. *Nat. Photonics* **1**, 473–478 (2007).

9 Faraji-Dana, M. *et al.* Compact folded metasurface spectrometer. *Nat. Commun.* **9**, 4196 (2018).

10 Sun, H. *et al.* In situ formed gradient bandgap-tunable perovskite for ultrahigh-speed color/spectrum-sensitive photodetectors via electron-donor control. *Adv. Mater.* **32**, 1908108 (2020).

11 Guo, L. *et al.* A single-dot perovskite spectrometer. *Adv. Mater.* **34**, 2200221 (2022).

12 Shinoda, K. & Ohtera, Y. Alignment-free filter array: Snapshot multispectral polarization imaging based on a Voronoi-like random photonic crystal filter. *Opt. Express* **28**, 38867-38882 (2020).

13 Grotevent, M. J. *et al.* Integrated photodetectors for compact Fourier-transform waveguide spectrometers. *Nat. Photonics* 17, 59–64 (2023).

14 Pohl, D. *et al.* An integrated broadband spectrometer on thin-film lithium niobate. *Nat. Photonics* **14**, 24–29 (2020).

15 Redding, B., Liew, S. F., Sarma, R. & Cao, H. Compact spectrometer based on a disordered photonic chip. *Nat. Photonics* **7**, 746–751 (2013).

16 Koshelev, A. *et al.* Combination of a spectrometer-on-chip and an array of Young’s interferometers for laser spectrum monitoring. *Opt. Lett.* **39**, 5645-5648 (2014).

17 Nitkowski, A., Chen, L. & Lipson, M. Cavity-enhanced on-chip absorption spectroscopy using microring resonators. *Opt. Express* **16**, 11930-11936 (2008).
